# Metabolomic Profiling of Mango (*Mangifera indica* Linn) Leaf Extract and Its Intestinal Protective Effect and Antioxidant Activity in Different Biological Models

**DOI:** 10.3390/molecules25215149

**Published:** 2020-11-05

**Authors:** Roberto O. Ybañez-Julca, Daniel Asunción-Alvarez, Ivan M. Quispe-Díaz, Javier Palacios, Jorge Bórquez, Mario J. Simirgiotis, Shagufta Perveen, Chukwuemeka R. Nwokocha, Fredi Cifuentes, Adrián Paredes

**Affiliations:** 1 Laboratorio de Farmacología, Facultad de Farmacia y Bioquímica, Universidad Nacional de Trujillo, Trujillo 13011, Peru; hasuncion@unitru.edu.pe (D.A.-A.); iquispe@unitru.edu.pe (I.M.Q.-D.); 2 Laboratorio de Bioquímica Aplicada, Facultad de Ciencias de la Salud, Universidad Arturo Prat, Iquique 1110939, Chile; 3 Laboratorio de Productos Naturales, Departamento de Química, Universidad de Antofagasta, Antofagasta 1270300, Chile; jorge.borquez@uantof.cl; 4 Instituto de Farmacia, Facultad de Ciencias, Universidad Austral de Chile, Valdivia 5110566, Chile; mario.simirgiotis@gmail.com; 5 Department of Pharmacognosy, College of Pharmacy, King Saud University, Riyadh 11451, Saudi Arabia; shakhan@ksu.edu.sa; 6 Department of Basic Medical Sciences, Faculty of Medical Sciences, The University of the West Indies, Mona Campus, Kingston 7, KGN, Jamaica; chukwuemeka.nwokocha@uwimona.edu.jm; 7 Laboratorio de Fisiología Experimental (EPhyL), Instituto Antofagasta (IA), Universidad de Antofagasta, Antofagasta 1270300, Chile; fredi.cifuentes@uantof.cl; 8 Departamento de Química, Facultad de Ciencias Básicas, Universidad de Antofagasta, Antofagasta 1271155, Chile; adrian.paredes@uantof.cl

**Keywords:** *Mangifera indica*, oxidative stress, relaxation, rat ileum, high-resolution orbitrap mass spectrometry, metabolome

## Abstract

*Mangifera indica* Linn popularly known as mango is used in folk medicine to treat gastrointestinal disorders. The aim of this study was to identify the metabolomic composition of lyophilized extract of mango leaf (MIE), to evaluate the antioxidant activity on several oxidative stress systems (DPPH, FRAP, TBARS, and ABTS), the spasmolytic and antispasmodic activity, and intestinal protective effect on oxidative stress induced by H_2_O_2_ in rat ileum. Twenty-nine metabolites were identified and characterized based on their ultra-high-performance liquid chromatography (UHPLC) high-resolution orbitrap mass spectrometry, these include: benzophenone derivatives, xanthones, phenolic acids, fatty acids, flavonoids and procyanidins. Extract demonstrated a high antioxidant activity in in-vitro assays. MIE relaxed (*p* < 0.001) intestinal segments of rat pre-contracted with acetylcholine (ACh) (10^−5^ M). Pre-incubation of intestinal segments with 100 µg/mL MIE significantly reduced (*p* < 0.001) the contraction to H_2_O_2_. Similar effects were observed with mangiferin and quercetin (10^−5^ M; *p* < 0.05) but not for gallic acid. Chronic treatment of rats with MIE (50 mg/kg) for 28 days significantly reduced (*p* < 0.001) the H_2_O_2_-induced contractions. MIE exhibited a strong antioxidant activity, spasmolytic and antispasmodic activity, which could contribute to its use as an alternative for the management of several intestinal diseases related to oxidative stress.

## 1. Introduction

*M. indica* is a tree of the Anacardiaceae family, and six varieties have been described: Kent, Keitt, Haden, Francis, Ataúlfo, and Tommy Atkins [1]. The distribution is mainly subtropical, with several metabolites reported to have been isolated and identified in the bark, seed, flowers, leaves, and pulp of mango [2], and the extracts are reported to have good antioxidant capacity [3].

Controversial reports as to the efficacy of use of medicinal plants, due to their antioxidant properties abounds [4]. Traditionally, *M. indica* leaves are used in colitis, diarrhea, and dysentery treatments [5,6]. The phytotherapeutic potential of mango could prevent intestinal damage, because it exhibits antioxidant and anti-inflammatory properties in several tissues [7], and its phytocomponents include: polyphenols, terpenes, sterols, carotenoids, vitamins, amino acids, etc. [8]. In a rat colitis model, the potential therapeutic anti-inflammatory/antioxidant activity of mango involves inhibition of cyclooxygenase (COX-1 and COX-2) by proanthocyanidin [9], inhibition via tumor necrosis factor (TNF)-α [10], IGF-1/mTOR pathway [11], as well as PI3K/Akt/mTOR pathway [7]. Therefore, oxidative stress, as an imbalance between the generation of reactive oxygen species (ROS) [12], could present with the increase of, and release of, pro-inflammatory cytokines, inflammation, and mucose damage in the intestine [13].

Consistent with the findings described above, we postulated that MIE has a spasmolytic, antispasmodic, and antioxidant effect on intestinal biological oxidative stress models.

First, we identified the metabolites of MIE using ultra-high-performance liquid chromatography (UHPLC) resolution mass spectrometry. HPLC profiling of compounds from mango is a good preliminary approach to determine functional value [14].

Second, to support the antispasmodic/antioxidant phytotherapy of mango, antioxidant activity on several oxidative stress systems (2,2-diphenyl-1-picryl-hydrazyl-hydrate, DPPH; Ferric Antioxidant Power, FRAP; Thiobarbituric Acid Reactive Substances, TBARS; and 2,2’-azino-bis(3-ethylbenzothiazoline-6-sulfonic acid), ABTS) was determined, followed by the intestinal protective effects on oxidative stress induced damage in rat ileum.

In this study, the H_2_O_2_ is used to produce an increase in the oxidative stress and intestinal motor contractions. Intestinal epithelial cells release a low level of H_2_O_2_ at nanomolar concentration [15]. H_2_O_2_ induces gastric motility in rats, as well as causes an increase in the contractile response of gastric fundic segments in a concentration-dependent manner [16]. In addition, hydrogen peroxide (H_2_O_2_) is a second messenger in metabolic processes with physiological functions [17].

Third, since H_2_O_2_-induced intestinal contractility reduces the cholinergic receptor response [18], we studied whether MIE would cause intestinal relaxation in pre-contracted tissue with acetylcholine (ACh) through modulation of potassium channels and Ca^2+^ influx from the extracellular space via cholinergic pathways.

## 2. Results

### 2.1. UHPLC-MS Metabolomic Analysis of MIE

**Figure 1:**
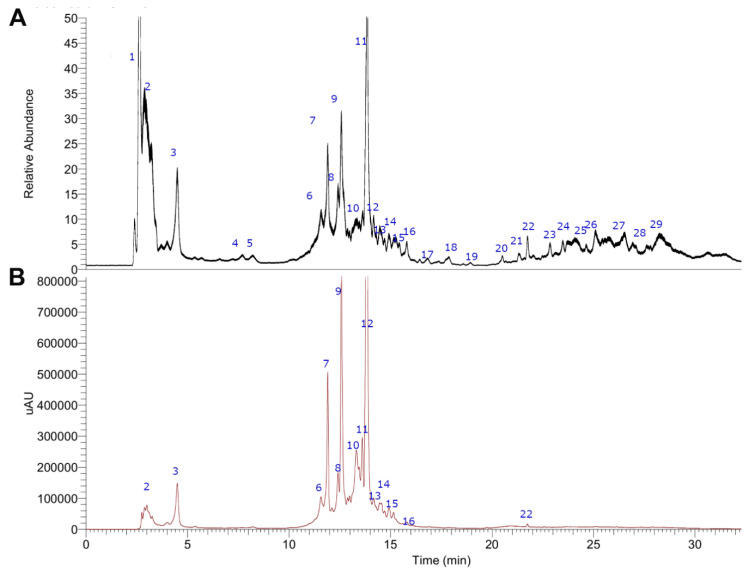
Ultra-high-performance liquid chromatography (UHPLC) chromatogram of MIE analyzed in positive ion mode. Total ion current (**A**), UV at 280 nm (**B**).

Twenty-nine compounds were identified in the chromatogram of the extract of MIE (Figure 1, Table 1). The spectra and structures of compounds detected are shown in Supplementary Materials Figure S1a–g. All compounds were already reported in mango or related plants, and compounds were characterized by accurate mass high molecular weights detection, some typical fragmentation patterns and UV spectra were obtained by Photodiode-Array (PDA) detector, plus literature reviews. The identification process is explained below.

#### 2.1.1. Benzophenone Derivatives

Compounds **6**–**8** and **14** were characterized as benzophenone derivatives (UV max at 236–279 nm). Peak **6** with a pseudomolecular ion at *m*/*z*: 407.09836 is tentatively identified as iriflophenone-3-C-β-d-glucoside (C_19_H_19_O_10_^−^) [23], while peak **8** with a pseudomolecular ion at *m*/*z*: 407.09833 as its isomer, iriflophenone-5-C-β-d-glucoside, and peak **7** as iriflophenone-3-C-β-d-galactoside. Peak **14** with a pseudomolecular ion at *m*/*z*: as 711.12140 as Iriflophenone-3-C-(2,3-di-O-galloyl)-β-d-glucoside (C_33_H_27_O_18_^−^) and peak **26** as skyrin (C_30_H_17_O_10_^−^). Biosynthetic relationship among the benzophenones detected in MIE is summarized in Figure S2 (Supplementary Materials).

#### 2.1.2. Xanthones Derivatives

Xanthones derivatives were peaks **11**–**13** and **16** [27]. Peak **11** with a pseudomolecular ion at *m*/*z*: 421.07773 as mangiferin (C_19_H_17_O_11_^−^) [22], while peak **12** with a pseudomolecular ion at *m*/*z*: 557.09387 as dehidro-mangiferin-6-O-gallate (C_26_H_21_O_14_^−^) and peak **13** as mangiferin-6-O-gallate (C_26_H_21_O_15_^−^), while peak **16** was identified as Iso mangiferin (C_19_H_17_O_11_^−^) and **25** as bellidin (C_13_H_8_O_6_^−^). Peak **9** exhibited signal at *m*/*z* 559.10938 [M + H]^+^ (C_26_H_23_O_14_) in addition to fragment ions at *m*/*z* 421 (C_19_H_17_O_11_) and *m*/*z* 305, indicative for the loss of protocatechuic acid and hexose sugar moieties, respectively (Figure S1b). The presence of mangiferitin as aglycone was confirmed from the fragment ion at *m*/*z* 305 [mangiferitin + 2Na]^+^, while the signal at *m*/*z* 421 annotated as mangiferin (C_19_H_17_O_11_). From the mass data of signal **7**, the structure tentatively identified as protocatechuic acid derivative of mangiferin (C_26_H_23_O_14_). To the best of our knowledge, this is the new secondary metabolite found in MIE. Previously mangiferin gallate has been identified in the pulp and peel of the mango fruits [22].

#### 2.1.3. Phenolic Acids

Peak **2** with a pseudomolecular ion at *m*/*z*: 191.05547 quinic acid (C_7_H_11_O_6_^−^), and peak **3** as citric acid (C_6_H_7_O_7_^−^), while peak **4** with a pseudomolecular ion at *m*/*z*: 169.01372 as gallic acid (C_7_H_5_O_5_^−^), and Peak **5** as gentisoyl glucoside (C_13_H_16_O_9_^−^). Peak **15** with a pseudomolecular ion at *m*/*z*: 197.04507 as syringic acid (C_9_H_9_O_5_^−^), peak **18** at *m*/*z*: 541.13489 sinapoyl-caffeoylshikimic acid (C_27_H_25_O_12_^−^), peak **20** as methyl gallate ester (C_16_H_13_O_9_^−^), and peak **21** with a pseudomolecular ion at *m*/*z*: 137.02379 as salicylic acid (C_7_H_5_O_3_^−^).

#### 2.1.4. Fatty Acids

Peaks **23** and **24** were tentatively identified as oxylipins, particularly, peak **23** with a pseudomolecular ion at *m*/*z*: 327.21790 as trihydroxyoctadienoic acid (C_18_H_31_O_5_^−^), and **24** as trihydroxyoctaenoic acid (C_18_H_33_O_5_^−^).

#### 2.1.5. Flavonoids

Peak **10** with a pseudomolecular ion at *m*/*z*: 577.11981 was identified as cicerin-7-malonylglucoside (C_26_H_25_O_15_^−^). Peak **17** displayed protonated molecular ion peak at *m*/*z* 447.09329 (C_21_H_19_O_11_) with fragment ion at *m*/*z* 271.15491 [M − 176]^+^, corresponding to the loss of a glucuronic acid moiety (176 amu). The presence of apigenin as aglycone connected to hexose sugar was also confirmed from fragment ion at *m*/*z* 271. Consequently, peak **17** was assigned as apigenin 7-O-glucuronide. Peak **19** as reynoutrin (C_20_H_18_O_11_^−^), **22** as quercetin (C_15_H_9_O_6_^−^), peak **28** as 5,8-dihydroxy-6,7,3-trimethoxy-3′,4′-methylenedioxyflavone (C_19_H_15_O_9_^−^), and, finally, peak **29** as eupatorin (C_18_H_15_O_7_^−^).

#### 2.1.6. Procyanidins

Peak **27** with a pseudomolecular ion at *m*/*z* 577.13544 was identified as procyanidin B1 (C_30_H_25_O_12_^−^). Others types of procyanidins dimers were previously described [27].

### 2.2. Total Phenolic Content and Antioxidant Activity of MIE

**Figure 2:**
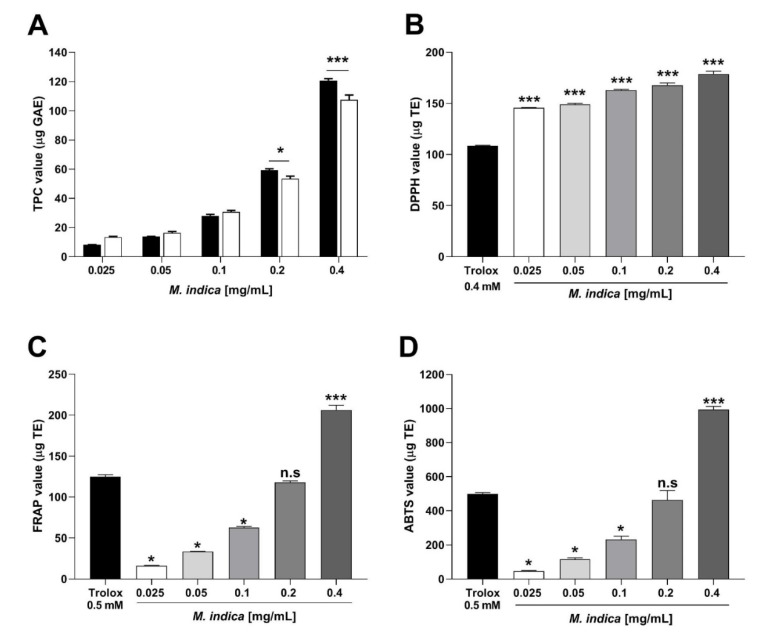
Total phenolic content (TPC) (**A**) and antioxidant activity of lyophilized extract of *M. indica* (MIE) by DPPH (**B**), FRAP (**C**), ABTS (**D**) assays. Panel (**A**), TPC was determined in an aqueous (black bar) and ethanolic (white bar) solution, while panels (**B**–**D**) show the aqueous solution of MIE. Panel (**A**) shows the data are expressed in micrograms of gallic acid equivalents (µg GAE), while, in panels (**B**–**D**), the data are expressed in micrograms of Trolox equivalents (µg TE). Each bar represents the mean ± the standard error of the mean (SEM) of three experiments (*n* = 3). n.s = not significant, statistical differences: * *p* < 0.05, *** *p* < 0.001 vs. black bar.

Phenolic compounds of MIE are responsible for the antioxidant activity. MIE re-suspended in distilled water (H_2_O) showed a concentration of total phenolic compounds (TPC) significantly (*p* < 0.001) higher than 96% ethanol (CH_3_CH_2_OH; Figure 2A). Gallic Acid Equivalents (GAE/mg) of extract was 279.875 µg for the dilution of 0.1 mg/mL of the extract. For the following experiments (antioxidant activity and acute experiments in isolated organ bath), the MIE was dissolved in distilled water.

Scavenger activity of DPPH radical of MIE (0.025, 0.05, 0.1, 0.2, and 4 mg/mL; 145.6 ± 0.3, 149 ± 1.1, 162.9 ± 0.8, 167.6 ± 2.3, and 178.7 ± 2.8 µg TE, respectively) was significantly (*p* < 0.001) better than Trolox 0.4 mM (108.6 ± 0.4 µg TE) as positive control (Figure 2B).

There were no statistically significant differences (*p* > 0.05) between the antioxidant activity of MIE (0.2 mg/mL) versus that of 0.5 mM Trolox according to the FRAP test (Figure 2C) and ABTS (Figure 2D). However, at a concentration of 0.4 mg/mL, MIE showed a significantly higher antioxidant activity (*p* < 0.001) than that of 0.5 mM Trolox according to FRAP (124.9 ± 2.4 µg TE control vs. 206.4 ± 5.97 µg TE with MIE; Figure 2C) and ABTS (497.6 ± 8.38 µg TE control vs. 993.9 ± 18.73 µg TE with MIE; Figure 2D).

### 2.3. Spasmolytic Activity of MIE on Rat Ileum

**Figure 3:**
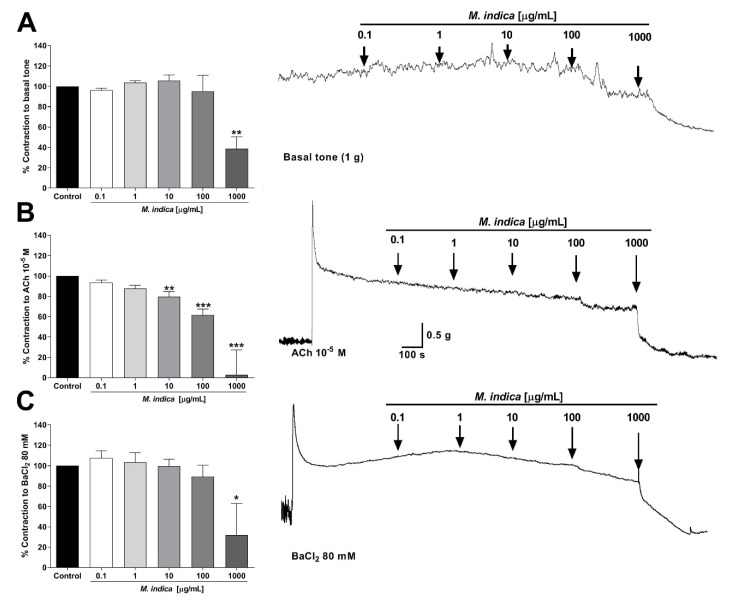
Spasmolytic activity of lyophilized extract of *M. indica* (MIE) in rat ileum. MIE generated a relaxation on the basal tone in ileal segments (**A**), MIE relaxed ileal segments pre-contracted with 10^−5^ M acetylcholine (ACh) (**B**), or 80 mM BaCl_2_ (**C**). In panel (**A**), control represents the basal tone (as 100% of contraction) without any treatment. In panels (**B**,**C**), control represents the maximum response (100%) induced by ACh and BaCl_2_, respectively. In addition, the original records of the relaxation effects of MIE in rat ileum are shown on the right side. Each bar represents the mean of response in percentage ± SEM of three experiments (*n* = 3). * *p* < 0.05; ** *p* < 0.01; *** *p* < 0.001 vs. control.

In order to evaluate the activity of MIE on the intestinal tone, ileal strips were used. MIE significantly decreased the muscle tone of rat ileum (39 ± 12% at 1000 µg/mL; *p* < 0.01) versus control (base tone) (Figure 3A).

To study the spasmolytic activity of MIE, intestinal strips were pre-contracted with ACh (muscarinic agonist) or BaCl_2_ (non-selective blocker of the current rectifying potassium channels; Kir). The extract significantly relaxed pre-contracted intestinal segments with 10^−5^ M ACh (80 ± 5% with 10 µg/mL extract; *p* < 0.01; Figure 3B), and with 80 mM BaCl_2_ compared to basal tone (32 ± 31% with 1000 µg/mL extract; *p* < 0.05; Figure 3C).

### 2.4. Antispasmodic Activity of MIE Reduced the Contractile Response to Acetylcholine in Rat Ileum: Role of Extracellular Calcium

**Figure 4:**
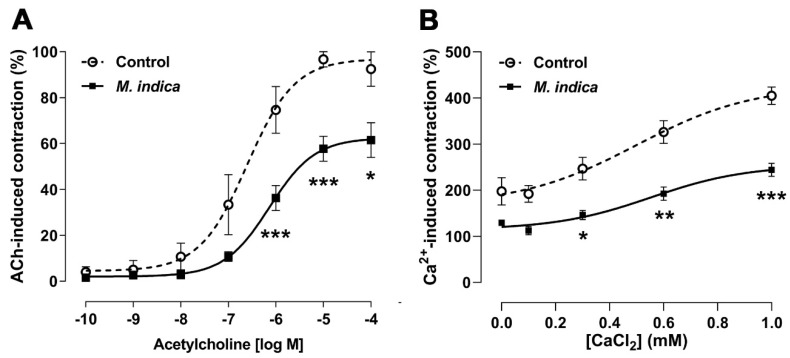
Antispasmodic activity of lyophilized extract of *M. indica* (MIE) reduces contractile response to acetylcholine (ACh). Rat ileum muscle tissue was pre-incubated with MIE (100 µg/mL) for 20 min before contraction with ACh (**A**) and pre-incubation with MIE 100 µg/mL reduced the influx of extracellular calcium (**B**). In both panels, control represents the contractile response of an ileal segment without pre-incubation with MIE (100 µg/mL). Each point represents the mean of maximal response in percentage ± SEM of three experiments (*n* = 3). * *p* < 0.05; ** *p* < 0.01; *** *p* < 0.001 vs. control.

This finding described above, was further clarified in ileal segments pre-incubated with MIE in organ bath. The extract (100 µg/mL) significantly reduced (*p* < 0.05) the maximum contraction to ACh (74 ± 10% control vs. 36 ± 5% with MIE; 10^−6^ M ACh; Figure 4A). The sensitivity (pIC_50_) to ACh in the presence of MIE (6.60 ± 0.18) was not significantly different to the control (6.14 ± 0.15).

Similar protocol was repeated with Ca^2+^-free medium, so as to evaluate the role of the Ca^2+^ influx in the contractile response to ACh. Firstly, the ileum segments were pre-incubated with MIE (100 µg/mL) for 20 min and then were stimulated with ACh (10^−5^ M) to induce a tonic contraction in the Ca^2+^-free medium. Second, the cumulative addition of extracellular calcium (0.1 mM to 1 mM) in the organ bath significantly increased the contractile response to ACh in the control (102 ± 5%) versus ileal strips pre-incubated with MIE (62 ± 4%; *p* < 0.001; Figure 4B). The sensitivity (pIC_50_) to ACh in the presence of MIE was not significantly different versus control.

### 2.5. MIE and Bioactive Molecules Attenuates the Acute Oxidative Stress Damage in Rat Ileum

The major constituents of the *M. indica* leaves include mangiferin, as well as gallic acid and quercetin [27]. Several researchers have demonstrated that *M. indica* leaves extract and their secondary metabolites, such as mangiferin, quercetin, and gallic acid, reduced the damage in colitis model in mice, as well as prevented oxidative and inflammatory effects [10,11,36,37].

**Figure 5:**
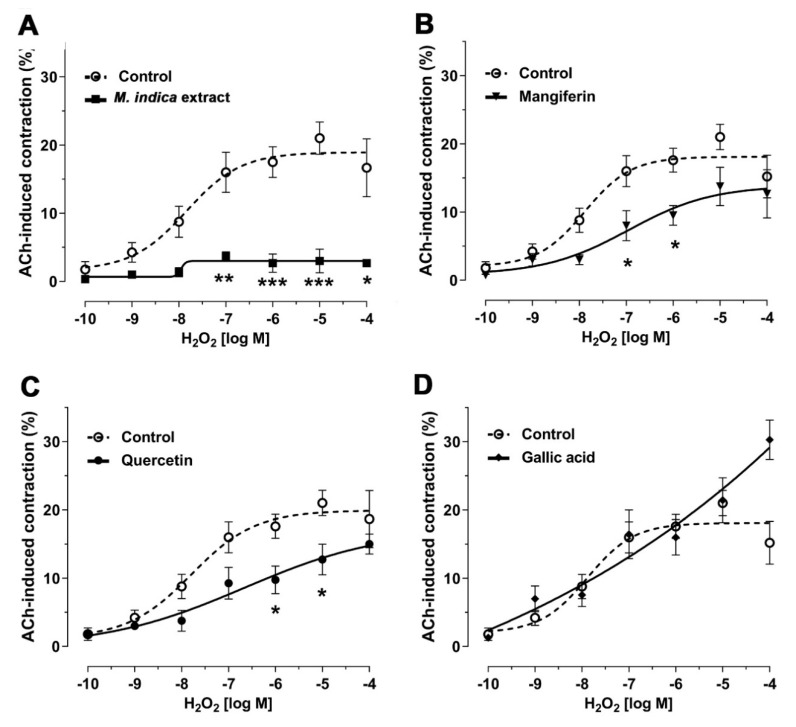
Antispasmodic activity of lyophilized extract of *M. indica* (MIE) and its main metabolites reduced contraction to H_2_O_2_. Rat ileum muscle tissue was pre-incubated with MIE (100 µg/mL; (**A**)), mangiferin (10^−5^ M; (**B**)), quercetin (10^−5^ M; (**C**)), or gallic acid (10^−5^ M; (**D**)) for 20 min before contraction with H_2_O_2_ (10^−10^ to 10^−4^). In all panels, control represents the contractile response to H_2_O_2_ of an ileal segment without pre-incubation with MIE, mangiferin, quercetin, or gallic acid, as appropriate. Each point represents the mean of maximal response in percentage ± SEM of five experiments (*n* = 5). * *p* < 0.05; ** *p* < 0.01; *** *p* < 0.001 vs. control.

To study wheher the antioxidant activity of MIE, and bioactive molecules, is associated with antispasmodic activity, intestinal strips from wild rats were pre-incubate with extract, mangiferin, quercetin, and gallic acid for 20 min before contraction with H_2_O_2_. MIE (100 µg/mL) significantly reduced the contraction to H_2_O_2_ (18 ± 2% control vs. 3 ± 1% with extract; 10^−6^ M H_2_O_2_; *p* < 0.001; Figure 5A). Similar results were observed for 10^−5^ M mangiferin (10 ± 1%; 10^−6^ M H_2_O_2_; *p* < 0.05; Figure 5B) and 10^−5^ M quercetin (10 ± 2%; 10^−6^ M H_2_O_2_; *p* < 0.05; Figure 5C) compared to control but not for gallic acid (Figure 5D). The sensitivities (pIC_50_) to H_2_O_2_ in the presence of MIE (7.98 ± 0.69), mangiferin (6.95 ± 0.60) and quercetin (6.67 ± 1.06) were not significantly different versus control (7.88 ± 0.29).

### 2.6. Chronic Treatment with MIE Reduced Ex-Vivo the Contractile Response to Acetylcholine and Acute Oxidative Stress Damage in Rat Ileum

**Figure 6:**
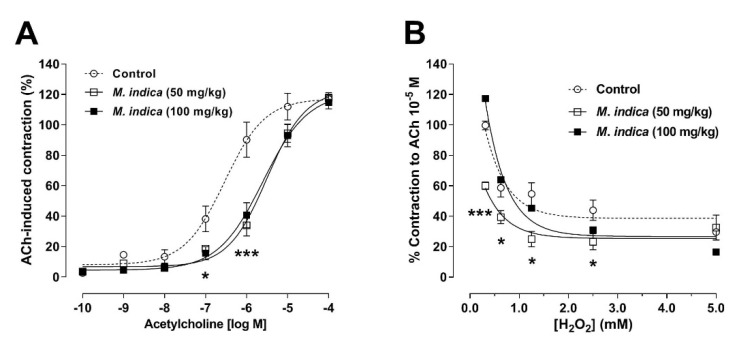
Chronic administration of lyophilized extract of *M. indica* (MIE) reduced contractile response to acetylcholine (**A**) and H_2_O_2_ (**B**). Treatment for 28 days with MIE 50 mg/kg (□) and 100 mg/kg (■) reduces the contractile response to ACh. Contractile response to ACh or H_2_O_2_ of the ileal segments of rats treated with peanut butter (vehicle) served as a control (**◯**). Each point represents the mean of maximal response in percentage ± SEM of five experiments (*n* = 5). * *p* < 0.05; *** *p* < 0.001 vs. control.

To confirm whether the acute effect observed by MIE on the contractile response to ACh (Figure 4A) would be replicated by chronic administration of the extract, groups of rats were orally treated with MIE for 28 days. The results showed that the ileal segments of the both treated-groups, 50 mg/kg and 100 mg/kg with MIE, significant decreased (*p* < 0.001) the contractile response to 10^−6^ M ACh versus control group (animals without treatment; Figure 6A): 90 ± 12% control vs. 41 ± 8% with 50 mg/kg MIE or 34 ± 7% with 100 mg/kg MIE. The sensitivity (pIC_50_) to ACh in the MIE-treated group significantly decreased (5.50 ± 0.09 with 50 mg/kg extract and 5.60 ± 0.12 with 100 mg/kg extract; *p* < 0.001) compared to the control group (6.54 ± 0.15).

The protective effect of MIE on H_2_O_2_-induced acute oxidative stress was evaluated in ileum from treated-rat for 28 days in organ bath. Firstly, the chronic treatment of animals with 50 mg/kg of MIE significantly (*p* < 0.001) reduced the contraction induced by 0.3125 mM H_2_O_2_ (99 ± 3% control vs. 39 ± 4% with 50 mg/kg MIE; Figure 6B). The sensitivity (pIC_50_) to H_2_O_2_ in the MIE group was not significantly different to control group.

**Figure 7:**
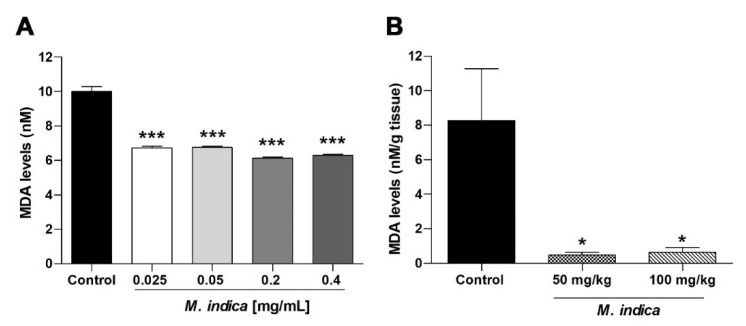
Lyophilized extract of *M. indica* (MIE) attenuates oxidative stress by reducing lipid peroxidation in-vitro and in-vivo. Panel (**A**) represent the test of TBARS for MIE in egg yolk homogenates, as a lipid-rich medium. In this case, the lipid peroxidation was induced by 2,2′-azobis(2-amidinopropane) dihydrochloride (0.07 M). Values represent the mean ± SEM of three experiments (*n* = 3). In panel (**B**), malondialdehyde (MDA) levels was significantly reduced in ileal homogenate in both group of rats treated with 50 mg/kg and 100 mg/kg of MIE. Values represent the mean of response in percentage ± SEM of five experiments (*n* = 5). * p < 0.05; *** *p* < 0.001 vs. control group.

Using a medium rich in lipids (egg yolk), we confirmed that the effect of MIE on the lipid peroxidation induced by 2,2′-azobis(2-amidinopropane) dihydrochloride (0.07 M). Figure 7A shows that at low concentrations MIE (0.025 mg/mL) is able to significantly reduce (*p* < 0.001) malondialdehyde (MDA) levels (10 ± 0.29 nM control vs. 6.72 ± 0.1 nM with MIE) according to the TBARS assay. Secondly, the effect on MDA levels in rat ileum homogenate was studied. It showed that chronic administration of MIE (50 mg/kg and 100 mg/kg) was able to significantly reduce (*p* < 0.05) lipid peroxidation: 8 ± 3 nM/g tissue control vs. 0.5 ± 0.2 nM/g tissue, 50 mg/kg MIE and 0.6 ± 0.3 nM/g tissue with 100 mg/kg MIE (Figure 7B).

## 3. Discussion

This is the first report that the oral supplementation with MIE protects intestinal tissue against oxidative damage, possibly because lyophilized MIE shows a high antioxidant activity. Mangiferin and quercetin may participate in the intestinal response in a similar way to the extract but not for gallic acid.

UHPLC chromatograph of MIE showed the presence of mangiferin, quercetin, gallic acid, vitamin C, and carotenoids which have a good capacity of capturing radicals [38]. The extract provided a high antiradical activity dose-dependently inhibiting the radical DPPH and ABTS at low concentrations, when compared with other studies [26,39,40]. Moreover, the FRAP assay also demonstrated that MIE has a high reducing power at low concentrations. It is known that phenolic, mangiferin, quercetin, carotenoid compounds, vitamin C, and gallic acid in mango are good donors of electrons that may reduce Fe^3+^ to Fe^2+^ [41]. The quantification of the content of phenolic (TPC) in MIE demonstrated that the extract contained high amounts of polyphenols. The TPC of the extract was higher than in previous studies from India [39], Thailand [40], Mauritius [26], and Brazil [21].

We postulated that antioxidant activity MIE has an antispasmodic and antioxidant effect on intestinal biological oxidative stress models. In order to gain insight on antispasmodic effect of MIE and antioxidant activity, intestinal contractility experiments were conducted in intestinal strips of rats pre-contracted with H_2_O_2_. We found that mangiferin and quercetin significant decreased the contraction to H_2_O_2_ in a similar way to the MIE but not for gallic acid. Lower concentrations of H_2_O_2_ were used in order to rule out the non-physiological effect of the high H_2_O_2_ concentrations, which goes beyond the enhancement of oxidative stress through promotion of ROS [42], and tonic contractions to upregulations of calcium uptake and cellular death [43]. Since mangiferin alone, or quercetin, did not mimic the effect of MIE, it is likely that synergic effect among bioactive molecules is necessary to carry out the effect of the extract.

Among the identified compounds of MIE with antioxidant activity include xanthones, such as mangiferin, mangiferin protocatechuic acid, quercetin and gallic acid [23,44]. Mangiferin, one of the main components identified in this study, is capable of improving the inflammatory bowel response and impaired gastrointestinal motility [45] because xanthone has a broad effect at the level of the small intestine [46].

We found that chronic supplementation of rats with MIE protected the tissues from oxidative damage induced by H_2_O_2_. The supplementation with MIE significantly reduced the contraction induced by H_2_O_2_ in the treated group. This result is in agreement with other reported studies that mango extract acts as exogenous antioxidant agent against oxidative stress damage in ovariectomized rats [23].

Furthermore, that supplementation with MIE decreased lipid peroxidation in the small intestine homogenate, in such way, protecting against oxidative damage induced by H_2_O_2_. This finding is consistent with our observation in homogenate of egg yolk, where we also confirmed that the extract decreased in-vitro lipid peroxidation by TBARS assay. This would be in agreement with other studies in bone and homogenized liver tissue, which reported that prolonged treatment of rats with *M. indica* prevents lipid peroxidation [23]. Our results strongly indicate that prolonged treatment of animals with MIE reduces oxidative stress in intestinal tissues and attenuates ex-vivo oxidative damage to intestinal function.

In a previous study, it was postulated that H_2_O_2_-induced intestinal contractility reduces the cholinergic receptors response [18]. Our study confirmed that MIE interfere with the intestinal contractile response mediated by the cholinergic pathway. MIE significantly relaxed the pre-contracted ileal segments with ACh and per se caused relaxation on the basal tone. MIE generates relaxation by blocking cholinergic receptors [47], histaminergic, or inhibition of leukotriene synthesis [48]. Thus, MIE relaxes the intestinal smooth muscle and would stimulate gastrointestinal transit through the cholinergic pathway [49].

This inhibitory mechanism of MIE on contractile response to ACh was mediated by the decrease in the influx of extracellular Ca^2+^. This finding is in agreement that mangiferin drastically reduced the contractile response to ACh in free Ca^2+^ medium and by extracellular Ca^2+^ addition in tracheal segments [50]. In this study, MIE also decreased the contractile response to BaCl_2_, a blocker of inward rectifier potassium channels (Kir). But, in addition to the role of Kir channels, the opening of Ca^2+^-dependent K^+^ channels (K_Ca_1.1) and the ATP-dependent potassium channels (K_ATP_) are involved in the antispasmodic effect of mango, as described in the smooth muscles of the trachea [50].

Oral administration of mangiferin accelerated gastro intestinal transit in mice involving cholinergic mechanism [49]. Antispasmodic activity of quercetin is through a cholinergic physiological mechanism [51]. Regarding gallic acid, it was reported that phenolic acids have antioxidant potential that attenuates the inflammation and ulcerative colitis [52]. These studies support our results on the intestinal antispasmodic effect of mangiferin and quercetin but not gallic acid.

Our results suggest that the antioxidant properties of MIE could contribute to counteract the ROS effect, when an imbalance of the endogenous antioxidant system of the cell occurs. In addition, the extract could readjust the unbalanced redox potential and pro-oxidant signaling systems in the cell, in such a way as to balance intracellular metabolism through regulation of receptors and ionic channels [53]. Thus, MIE would cause relaxation of the intestinal tissue by inhibition of the cholinergic receptor. This mechanism may involve the opening of K^+^ channels, which leads to a decrease of Ca^2+^ influx from extracellular space and a decrease in gastrointestinal motility [54]. These findings would be beneficial because the small intestine has fewer enzymatic and non-enzymatic protective factors against oxidative stress compared to the large intestine [55].

## 4. Material and Methods

### 4.1. Chemicals

Acetylcholine hydrochloride (ACh), 1,1-diphenyl-2-picrylhydrazyl radical (DPPH), 2,2′-azino-bis(3-ethylbenzothiazoline-6-sulfonic acid) diammonium salt (ABTS), Folin & Ciocalteu’s phenol reagent, sodium bicarbonate (NaHCO_3_), (±)-6-hydroxy-2,5,7,8-tetramethylchromane-2-carboxylic acid (Trolox), potassium persulfate (K_2_S_2_O_8_), quercetin, and gallic acid were purchased from Sigma-Aldrich (St. Louis, MO, USA). Mangiferin was provided by Dr. Gabino Garrido and characterized by Dr. Alberto Núñez in a previous study [56]. 2-thiobarbituric acid (TBA), 1,1,3,3-tetraethoxypropane (MDA), 2,4,6-tris(2-pyridyl)-s-triazine (TPTZ), 2,2′-azobis(2-amidinopropane) dihydrochloride (AAPH), sodium carbonate (Na_2_CO_3_), sodium acetate, pyridine, iron (III) chloride hexahydrate (FeCl_3_**·**H_2_O), ethylenediaminetetraacetic acid (EDTA), hydrochloric acid (HCl), sodium chloride (NaCl), potassium chloride (KCl), magnesium chloride (MgCl_2_), sodium phosphate monobasic (NaH_2_PO_4_), barium chloride (BaCl_2_), and calcium chloride (CaCl_2_) were from Merck (Peruana S. A, Ate, Lima, Perú). Trichloroacetic acid (TCA) was from Fisher Chemical (Allentown, PA, USA), sodium dodecyl sulfate (SDS) was from ICN Biochemical Inc. (Cleveland, OH, USA), n-butanol and d-glucose were from Riedel-de Haën (Seelze, Germany), and hydrogen peroxide (H_2_O_2_) solution was prepared from a commercial product (Laboratorio Alkofarma, Lima, Perú).

### 4.2. Plant Material

*M. indica* L. cv. Kent (5 kg) leaves were collected in the district of Bagua Grande, province of Utcubamba, Amazonas region in Peru; with coordinates of south latitude 5°45′26″; West longitude 78°26′43″ and altitude of 450 m above mean sea level. The identification was made in the *Herbarium Truxillense* of the National University of Trujillo and was assigned the identification code HUT 59581. The fresh leaves were washed, dissected, crushed, and then extracted using a 50% hydroalcoholic (H_2_O:CH_3_CH_2_OH; 1:1) reflux system. After two hours, it was filtered with the help of a vacuum, and the extract was then concentrated by evaporation (Heidolph, Schwabach, Germany). The concentrated extract was resuspended in water, then taken to a Shell Freezer (Labconco, Kansas City, MO, USA) and frozen at −80 °C (Arctiko, Esbjerg, Denmark) to subsequently be lyophilized (Millrock, NY, USA). The lyophilizates were kept refrigerated at 4 °C in hermetically sealed Falcon tubes with parafilm.

### 4.3. UHPLC-DAD-MS Instrument and LC Parameters and MS Parameters

A Thermo UHPLC Dionex Ultimate 3000 system (Thermo Fisher Scientific, Darmstadt, Germany) hyphenated with a Thermo Q Exactive focus machine (Thermo Fisher Scientific, Darmstadt, Germany) was used [57]. For the analysis, 5 mg of MIE were dissolved in 2 mL of methanol, filtered through a 200 µm polytetrafluoroethylene (PTFE) filter. Ten microliters was injected into the instrument, with all specifications set as reported [57]. Liquid chromatography was performed using an UHPLC C18 column (Acclaim, 150 mm × 4.6 mm ID, 2.5 µm, Thermo Fisher Scientific, Bremen, Germany) operated at 25 °C. The detection wavelengths were 280, 254, 330, and 354 nm, and photodiode array detectors (Thermo Fisher Scientific, Darmstadt, Germany) were set from 200 to 800 nm. Mobile phases were 1% formic aqueous solution (A) and acetonitrile 1% formic acid (B). The gradient program time was started at 5% B at zero time, then maintained 5% B for 5 min, then going to 30% B for 10 min, then maintaining 30% B for 15 min, then going to 70% B for 5 min, then maintaining 70% B for 10 min, and, finally, coming back to initial conditions in 10 min, with 12 min for column equilibration before each injection. The flow rate was 1 mL per min, and the injection volume was 10 µL. Standards and the lyophilized decoction dissolved in methanol were kept at 10 °C during storage in the autosampler. Detection of all compounds was performed using a Q-Exactive Orbitrap mass spectrometer (Thermo, Bremen, Germany) at 17,500 full width half Maximum (FWHM) (*m*/*z* 200), and the Heated Electrospray Ionization Source II (HESI II) probe values were optimized as previously described [57].

### 4.4. Determination of Antioxidant Activity

In-vitro antioxidant activity was determined using the methods described in Supplementary Materials. Several assays, such as total phenolic content (TPC), DPPH, TBARS, FRAP, and ABTS were used to evaluate the MIE. The absorbance of each assay was determined in a microplate reader (AccuSkan GO UV/Vis; Fisher Scientific; Allentown, PA, USA).

### 4.5. Animals 

The experiments in this study were carried out following the procedures of the American Veterinary Medical Association (AVMA) and the Ethics Committee of Pharmacy and Biochemistry Faculty of the National University of Trujillo (COD.N°: P 012-19/CEIFYB). Twenty male *Rattus norvegicus* Holtzman (8–10 weeks old and 170 g to 200 g) were used. They remained in their boxes at a temperature of 22–25 °C, with 12 h light/dark cycles, and were fed with standard rat chow (Molinorte S.A.C., Trujillo, Perú) and water ad libitum. Five animals were used for acute protocols and fifteen were used for chronic protocols.

### 4.6. Study of the Chronic Administration of MIE on the Cholinergic Response and Acute Oxidative Stress Induced by H_2_O_2_

Holtzman rats were randomly distributed in 3 experimental groups as follows:

- Group 1 (*n* = 5; Control) treated with vehicle (peanut butter; ConAgra Foods Export Company Inc., Omaha, NE, 68102, USA).
- Group 2 (*n* = 5; MIE 50 mg/kg) treated with MIE (50 mg/kg) plus vehicle for 28 days.
- Group 3 (*n* = 5; MIE 100 mg/kg) treated with MIE (100 mg/kg) plus vehicle for 28 days.

Both peanut butter (vehicle) and MIE were orally administered every day for 28 days on a spatula. At the end of the experiment regimen, all groups were sacrificed, and the ileal samples were prepared and taken to the isolated organ bath for ex-vivo contractile reactivity study in the presence of ACh (10^−10^ M to 10^−4^ M) and H_2_O_2_ (0.3125 mM to 5 mM). In addition, lipid peroxidation levels were also determined by quantifying the malondyaldehide (MDA) levels in ileum homogenate.

### 4.7. Intestinal Reactivity Experiments

Each rat was sacrificed by cervical dislocation. A portion of ileum (2.5 cm), without considering the 10 cm nearest to the ileocecal valve, was removed and placed in a petri dish which contained Tyrode solution; this solution had the following composition (in 10^−3^ M): NaCl 136.9; KCl 2.68; CaCl_2_ 1.8; MgCl_2_ 1.05; NaHCO_3_ 11.9; NaH_2_PO_4_ 0.42, and d-glucose 5.55. The resting tension was fixed at 1 g. The experimental data was recorded through a Power Lab 26T system (ADInstruments Pty Ltd, New South Wales, Australia) with the Chart v5.5 program for Windows (MLS013/W, Colorado Springs, Colorado, CO, USA). Acute protocols with MIE were as follows: relaxation in basal tone, in pre-contracted rat ileum with BaCl_2_ (80 mM) and Acetylcholine (Ach, 10^−5^ M), reduction of the contractile response to ACh (10^−5^ M) in normal Ca^2+^ and free Ca^2+^ physiological solution. In addition, the relaxation effect of mangiferin, quercetin, and gallic acid in tissue with cumulative concentrations of H_2_O_2_ was evaluated. These assays were described in detail in Supplementary Materials.

### 4.8. Determination of Lipid Peroxidation in Ileum by TBARS

The test was performed according to the method published previously [58] with slight modifications. The tissues were homogenized and centrifuged. Then, the supernatant was read at 532 nm. Data from five experiments were presented as nanomoles (10^−9^ M) of malondyaldehide (MDA) per gram of tissue.

### 4.9. Statistical Analysis

GraphPad Prism 8.0.2 software (San Diego, CA, USA) was used for statistical analysis. To compare dose-response curves, non-linear regression curves were performed, and, for the evaluation of the significance between groups, two-way ANOVA was used as appropriate, followed by the Bonferroni test as a post hoc test. In addition, the half maximal inhibitory concentration (pIC_50_) was calculated by nonlinear regression (sigmoidal). One-way ANOVA was used to compare the significant differences between several groups of antioxidant activity. *p* < 0.05 was considered statistically significant.

## 5. Conclusions

The extract for the species *M. indica* showed significant intestinal relaxation. This effect could be attributed to the presence of 29 compounds detected by UHPLC high-resolution orbitrap mass spectrometry. Some of these bioactive molecules include: benzophenone derivatives, xanthones, phenolic acids, fatty acids, flavonoids, and procyanidins.

Since MIE caused intestinal relaxation by inhibition of the cholinergic receptor, involving K^+^ channels and decrease of Ca^2+^ influx from extracellular in rat ileum, and the compounds mangiferin and quercetin also caused intestinal relaxation similar to MIE, these compounds, to some extent, are likely responsible for intestinal relaxation. However, the synergistic effect could not rule out, and more research is needed to support the antispasmodic effects of the bioactive molecules present in this extract. The antioxidant properties of MIE, together with its antispasmodic effect on oxidative stress induced responses, would be an alternative to optimize the treatment of diseases, such as irritable and inflamed bowel syndrome, where current drugs have not been very successful. In future studies, it would be interesting to investigate in-vivo effects of MIE on the production of endogenous peroxides, as well as the molecular mechanism underlying the contraction induced by oxidative stress.

## Figures and Tables

**Table 1:** High resolution UHPLC-Photodiode Array Quadrupole Orbitrap (PDA-Q-OT) identification of metabolites in MIE.

Peak #	Retention Time (min.)	UV Max	Tentative Identification	Molecular Formula	Theoretical Mass (*m*/*z*)	Measured Mass (*m*/*z*)	Accuracy (δ ppm)	References	MS^2^ Ions (*m*/*z*)
1	2.40		unknown			272.9586		-	
2	3.22	-	Quinic acid	C_7_H_11_O_6_^−^	191.05611	191.05547	5.10	[19]	127.03929, 85.02829
3	4.51	235	Citric acid	C_6_H_7_O_7_^−^	191.01973	191.01936	3.01	[19,20]	111.00771 C_5_H_3_O_3_^−^
4	8.21	236–271	Gallic acid	C_7_H_5_O_5_^−^	169.01425	169.01372	3.20	[19,21,22]	125.02363 C_6_H_5_O_3_^−^ [M^−^ − CO_2_]
5	8.70		Gentisoyl glucoside	C_13_H_16_O_9_^−^	315.07106	315.07227	3.83	PubChem 101339724	287.05600, 153.08177
6	11.25	236–294	Iriflophenone-3-C-β-d-glucoside	C_19_H_19_O_10_^−^	407.09837	407.09837	2.69	[19,21,23]	117.03393 C_6_H_5_O_3_^−^
7	11.95	236–294	Iriflophenone-3-C-β-d-galactoside	C_19_H_19_O_10_^−^	407.09837	407.09836	2.66	[21,23]	117.03393 C_6_H_5_O_3_^−^
8	12.32	236–294	Iriflophenone-5-C-β-d-glucoside	C_19_H_19_O_10_^−^	407.09837	407.09833	2.65	[21,23]	125.02370 C_6_H_5_O_3_^−^
9	12.54	236–277	Gallic acid derivative of iriflophenone	C_26_H_23_O_14_	559.10933	559.10883	−0.89	[21]	421.07785 C_19_H_17_O_11_
10	13.43	280	Cicerin 7 (6′-malonyl) glucoside	C_26_H_25_O_15_^−^	577.11880	577.11981	1.75	[24]	179.05022, 151.00436
11	13.72	258–318	Mangiferin	C_19_H_17_O_11_^−^	421.07763	421.07773	2.82	[21,22,23,25,26]	258.01666 C_13_H_6_O_6_^−^ [M^−^ − glucose]
12	14.02	236–274	Dehidro-mangiferin-6-O-gallate	C_26_H_21_O_14_^−^	557.09368	557.09387	2.53	[27]	303.09067
13	14.21	236–260	Mangiferin-6-O-gallate	C_26_H_21_O_15_^−^	573.08859	573.08820	−0.68	[21,25,26]	421.07762 C_19_H_17_O_11_^−^
14	14.35	236–279	Iriflophenone-3-C-(2,3-di-O-galloyl)-β-d-glucoside	C_33_H_27_O_18_^−^	711.12029	711.12140	4.65	[21,25]	245.21232
15	15.02	238–271	Syringic acid	C_9_H_9_O_5_^−^	197.04555	197.04507	3.16	[19]	124.01559 C_6_H_4_O_3_^−^ [M^−^ − CO_2_ − 2Me]
16	16.32	258–318	Iso mangiferin	C_19_H_17_O_11_^−^	421.07763	421.07776	2.89	[21,25,26]	258.01666 C_13_H_6_O_6_^−^ [M^−^ − glucose]
17	16.83	237–269	Apigenin 7-O-glucuronide	C_21_H_18_O_11_	447.09329	447.09280	2.26	[25]	271.15491, 225.05186, 179.0765, 150.9982
18	17.35	238–271	Sinapoyl-caffeoylshikimic acid	C_27_H_25_O_12_^−^	541.13515	541.13489	1.43	[28]	507.06539, 463.25494
19	18.92	254–354	Reynoutrin	C_20_H_18_O_11_^−^	477.07654	433.07767	2.60	[29]	179.05022, 151.00436
20	20.55	220–280	OMe-gallic acid/methyl gallate ester	C_16_H_13_O_9_^−^	349.05651	349.05661	3.35	[21]	125.05342
21	21.37	220–265	Salicylic acid	C_7_H_5_O_3_^−^	137.02442	137.02379	3.25	[22]	93.03452, 59.01385
22	21.75	254–354	Quercetin	C_15_H_9_O_6_^−^	301.03538	301.03546	4.02	[26]	179.05012, 151.0023
23	23.23	215	Trihydroxyoctadienoic acid (Trihydroxylinoleic acid)	C_18_H_31_O_5_^−^	327.21770	327.21790	3.95	[30]	259.06116, 174.95134
24	23.76	209	Trihydroxyoctaenoic acid	C_18_H_33_O_5_^−^	329.23335	329.23349	3.85	[30]	293.17896, 239.09236,
25	24.23	258–318	Bellidin (1,3,5,8-tetrahydroxyxanthone)	C_13_H_8_O_6_^−^	259.02371	259.02475	3.99	[31,32]	197.04517, 174.95560
26	25.12	258–318	Skyrin	C_30_H_17_O_10_^−^	537.08162	537.08264	1.89	[33]	387.07208, 325.20193
27	26.45	280	Procyanidin B1	C_30_H_25_O_12_^−^	577.13405	577.13544	2.39	[34]	407.07702, 289.07120, 125.02291
28	27.20	280	5,8-dihydroxy-6,7,3-trimethoxy-3′,4′-methylenedioxyflavone	C_19_H_15_O_9_^−^	387.07106	387.07236	−1.25	PubChem NSC678101	179.05012, 151.0023
29	28.80	254–354	Eupatorin	C_18_H_15_O_7_^−^	343.08123	343.08258	3.93	[35]	179.05026, 151.0045

## Supplementary Materials

The following are available online, Figure S1a–g: Full MS spectra and structures of peaks **6**, **9**, **11**, **17**, **25**, **26**, and **27**; Figure S2: Biosynthetic benzophenones. Methods for Intestinal reactivity experiments and the determination of antioxidant activity: TPC, DPPH, TBARS, FRAP, and ABTS.
