# Supplementary material for: Metabolomic Profiling of Mango (Mangifera indica Linn) Leaf Extract and Its Intestinal Protective Effect and Antioxidant Activity in Different Biological Models
Source: Molecules. 2020 Nov 5;25(21):5149. doi: 10.3390/molecules25215149 (PMC7663946; doi:10.3390/molecules25215149)
Supplement: Supplementary file 1 [file molecules-25-05149-s001.pdf]

Supplementary material for the article:

Metabolomic Profiling of Mango (*Mangifera indica* Linn) Leaf Extract and Its Intestinal Protective Effect and Antioxidant Activity in Different Biological Models

Roberto O. Ybañez-Julca [1,*], Daniel Asunción-Alvarez [1], Ivan M. Quispe-Díaz [1], Javier Palacios [2,*], Jorge Bórquez [3], Mario J. Simirgiotis [4], Shagufta Perveen [5], Chukwuemeka R. Nwokocha [6], Fredi Cifuentes [7] and Adrián Paredes [8]

[1] Laboratorio de Farmacología, Facultad de Farmacia y Bioquímica, Universidad Nacional de Trujillo, Trujillo 13011, Perú; hasuncion@unitru.edu.pe (D.A.-A.); iquispe@unitru.edu.pe (I.M.Q.-D.)
[2] Laboratorio de Bioquímica Aplicada, Facultad de Ciencias de la Salud, Universidad Arturo Prat, Iquique 1110939, Chile
[3] Laboratorio de Productos Naturales, Departamento de Química, Universidad de Antofagasta, Antofagasta 1270300, Chile; jorge.borquez@uantof.cl (J.B.)
[4] Instituto de Farmacia, Facultad de Ciencias, Universidad Austral de Chile, Valdivia 5110566, Chile; mario.simirgiotis@gmail.com (M.J.S)
[5] Department of Pharmacognosy, College of Pharmacy, King Saud University, Riyadh 11451, Saudi Arabia; shakhan@ksu.edu.sa (S.P)
[6] Department of Basic Medical Sciences, Faculty of Medical Sciences, The University of the West Indies, Mona Campus, Kingston 7, KGN, Jamaica; chukwuemeka.nwokocha@uwimona.edu.jm (C.R.N)
[7] Laboratorio de Fisiología Experimental (EPhyL), Instituto Antofagasta (IA), Universidad de Antofagasta, Antofagasta 1270300, Chile; fredi.cifuentes@uantof.cl (F.C)
[8] Departamento de Química, Facultad de Ciencias Básicas, Universidad de Antofagasta, Antofagasta 1271155, Chile; adrian.paredes@uantof.cl (A.P)
* Correspondence: rybanez@unitru.edu.pe (R.O.Y.-J.); clpalaci@unap.cl (J.P.); Tel: +51-0449-7634-5993 (R.O.Y.-J.); +56-57-252-6910 (J.P.)

Figure S1 (a-g): Full MS spectra and structures of peaks 6 (a), 9 (b), 11 (c), 17 (d), 25 (e), 26 (f), 27 (g).

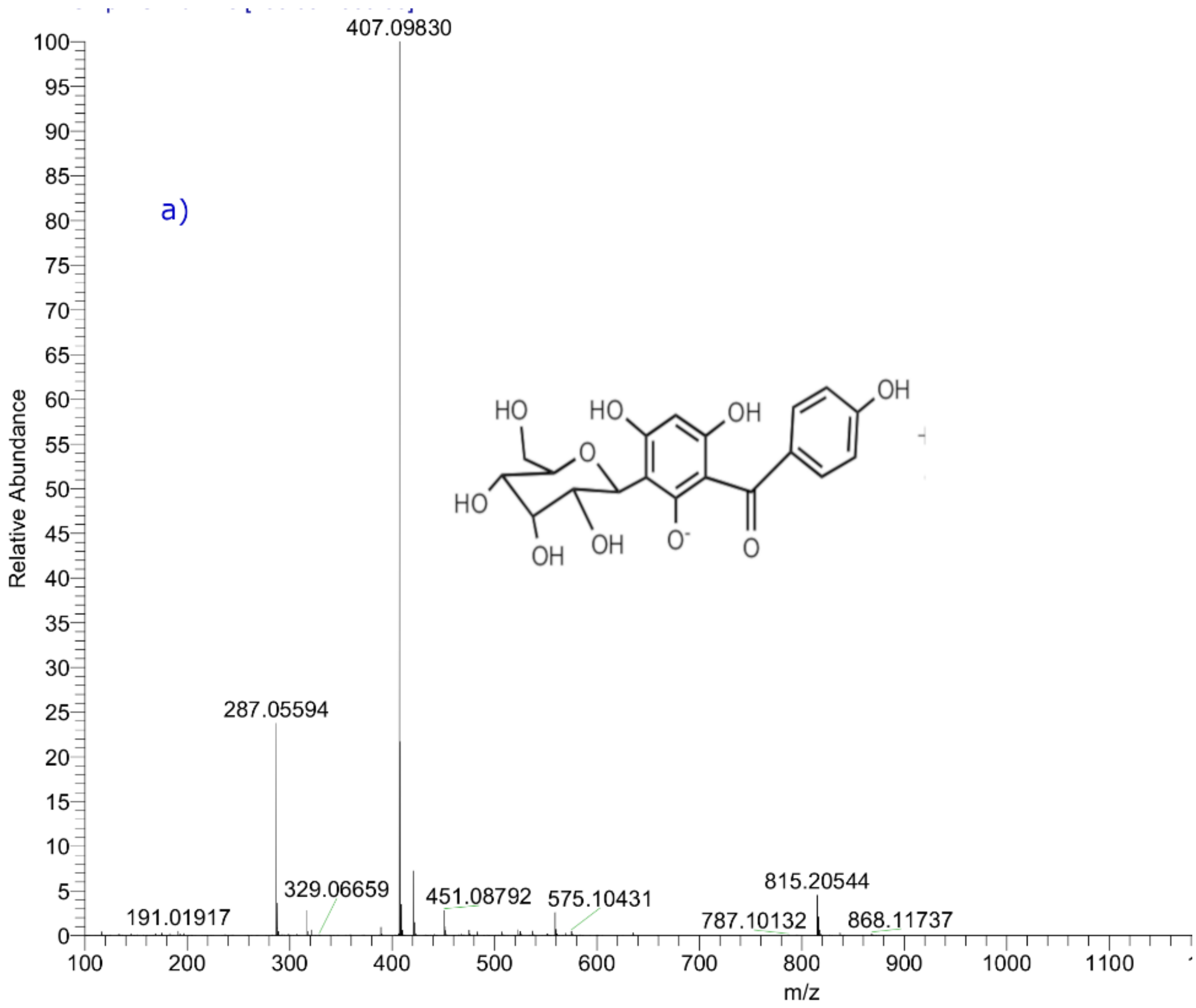

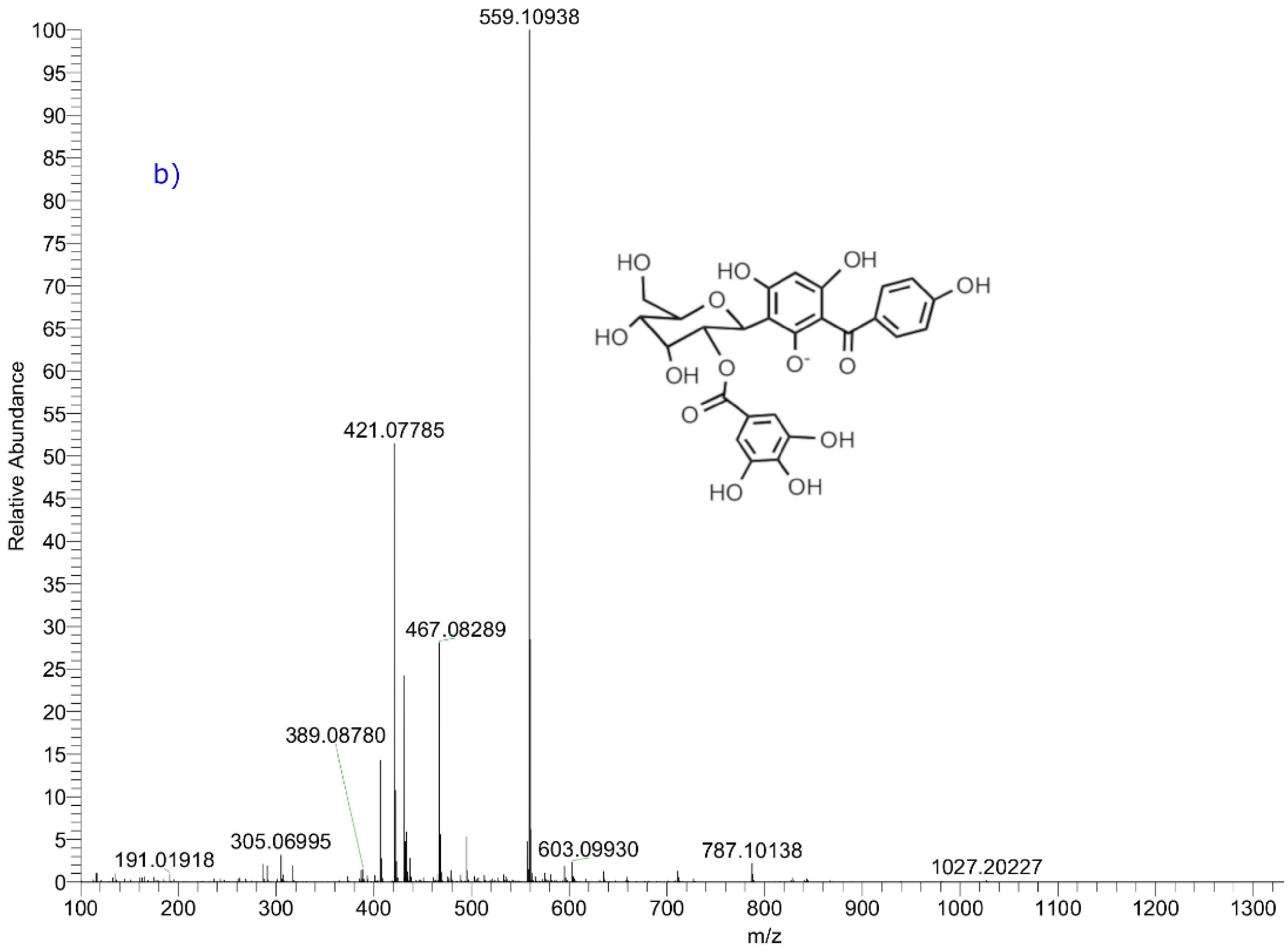

b)
559.10938
421.07785
467.08289
389.08780
305.06995
191.01918
603.09930
787.10138
1027.20227
Relative Abundance
m/z
HO
HO
OH
OH
O
HO
OH
O
O⁻
O
O
OH
HO
OH

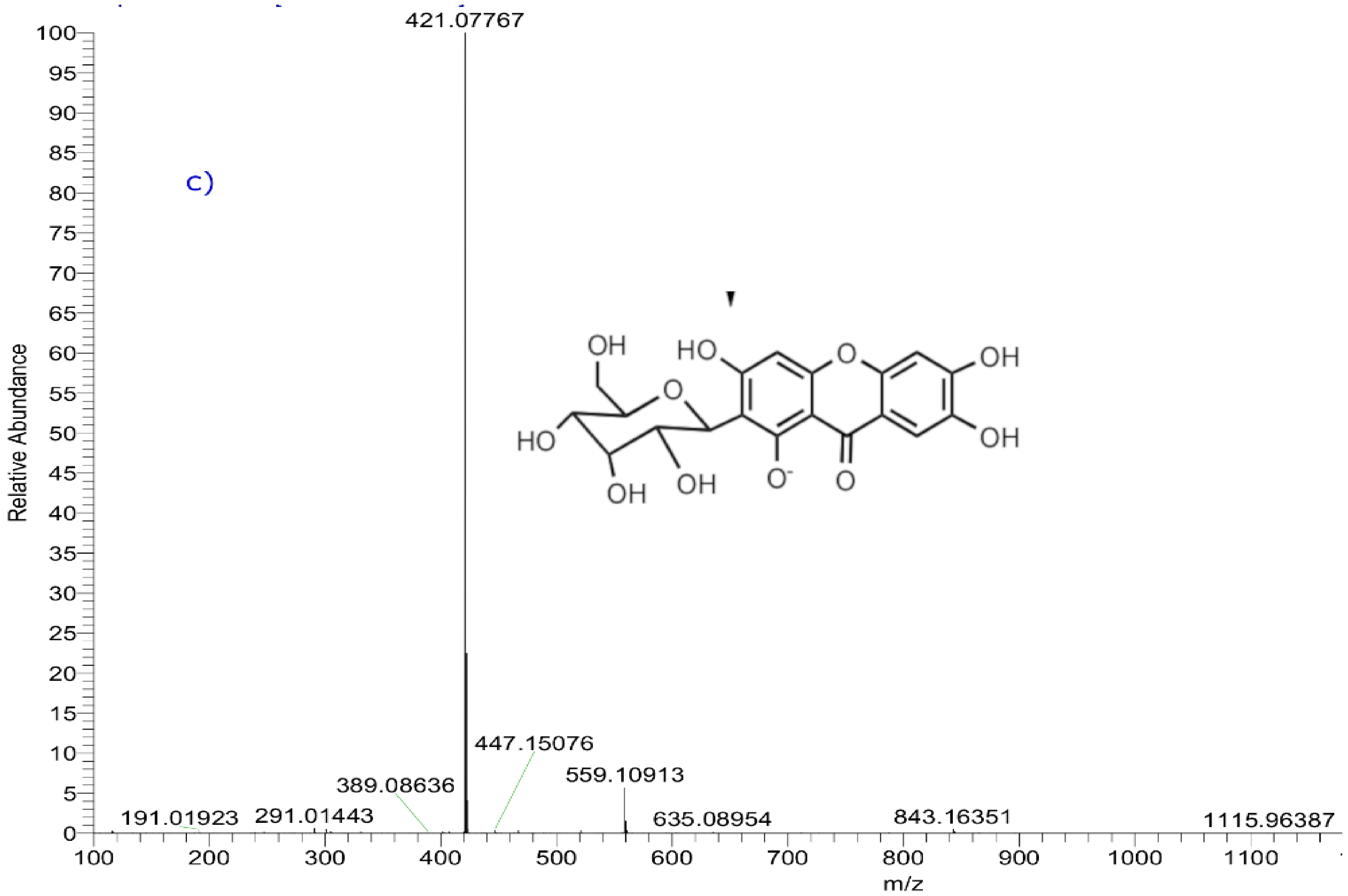

c)
421.07767
447.15076
389.08636
559.10913
191.01923
291.01443
635.08954
843.16351
1115.96387
Relative Abundance
m/z
100
95
90
85
80
75
70
65
60
55
50
45
40
35
30
25
20
15
10
5
0
100
200
300
400
500
600
700
800
900
1000
1100
OH
HO
O
OH
HO
OH
OH
O
OH
OH
O

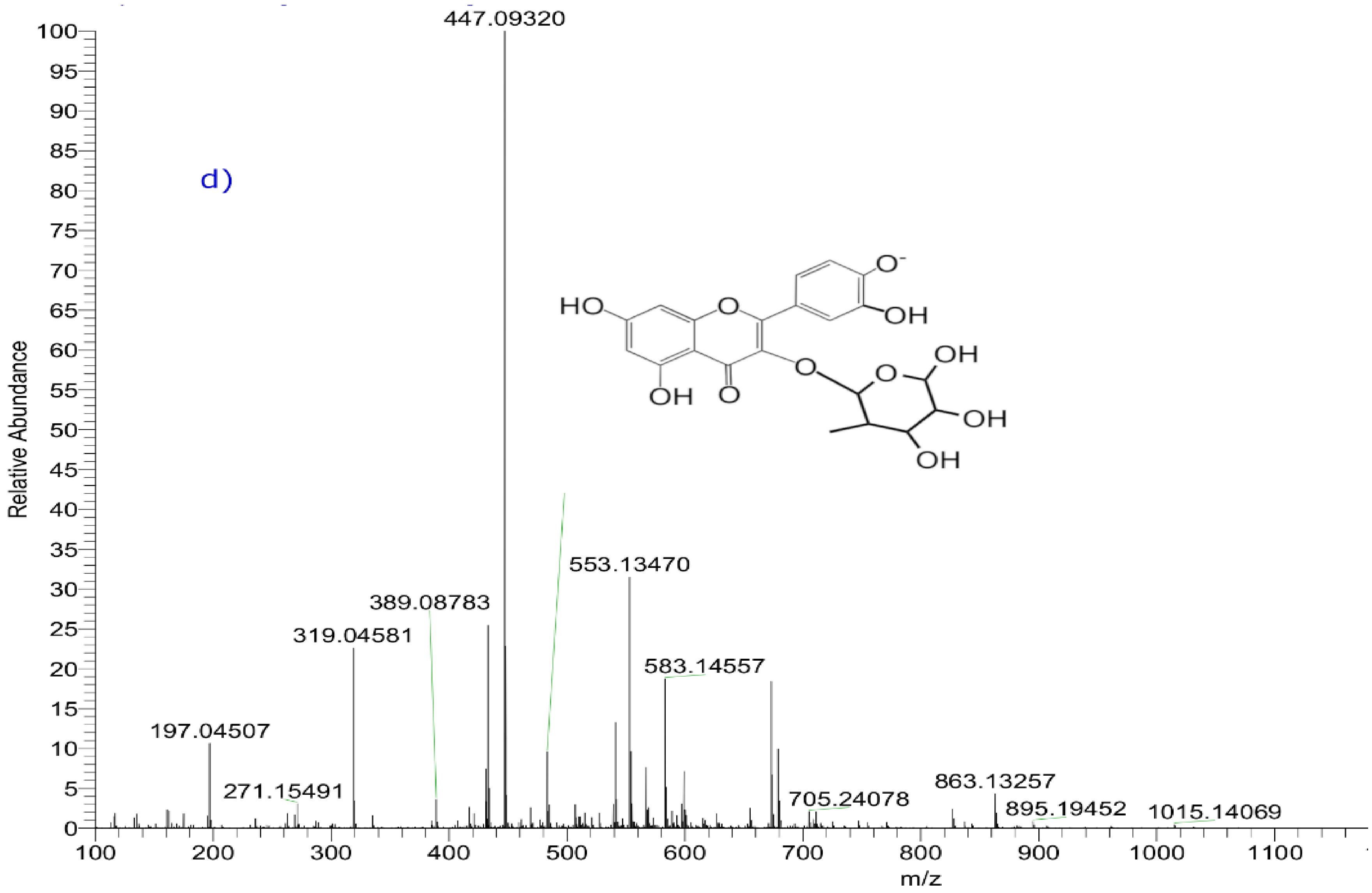

d)
447.09320
553.13470
389.08783
319.04581
583.14557
197.04507
271.15491
705.24078
863.13257
895.19452
1015.14069
Relative Abundance
m/z
HO
O
O
OH
O⁻
OH
O
O
OH
OH
OH

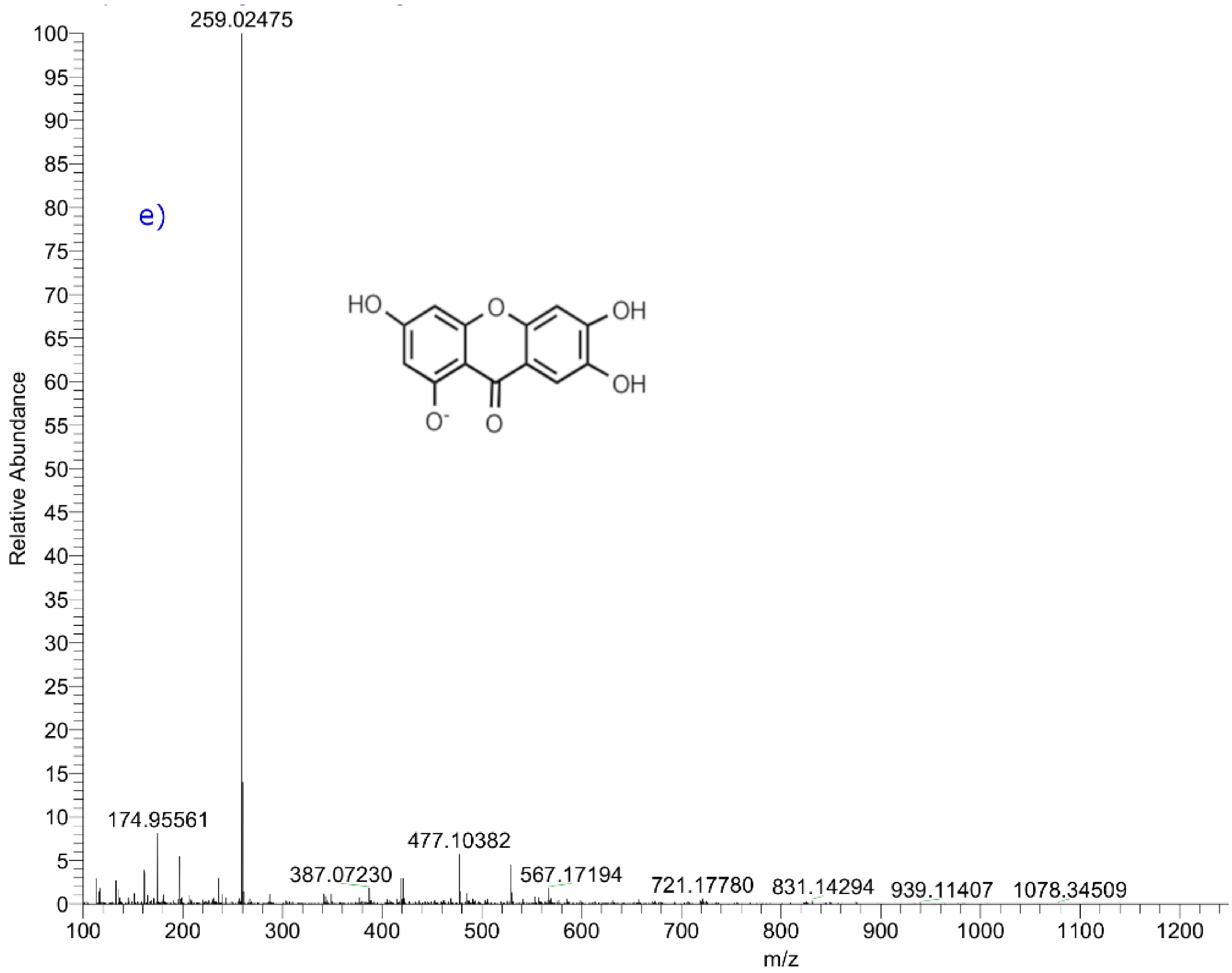

e)
259.02475
174.95561
477.10382
387.07230
567.17194
721.17780
831.14294
939.11407
1078.34509
HO
O
OH
OH
O-
O
Relative Abundance
m/z

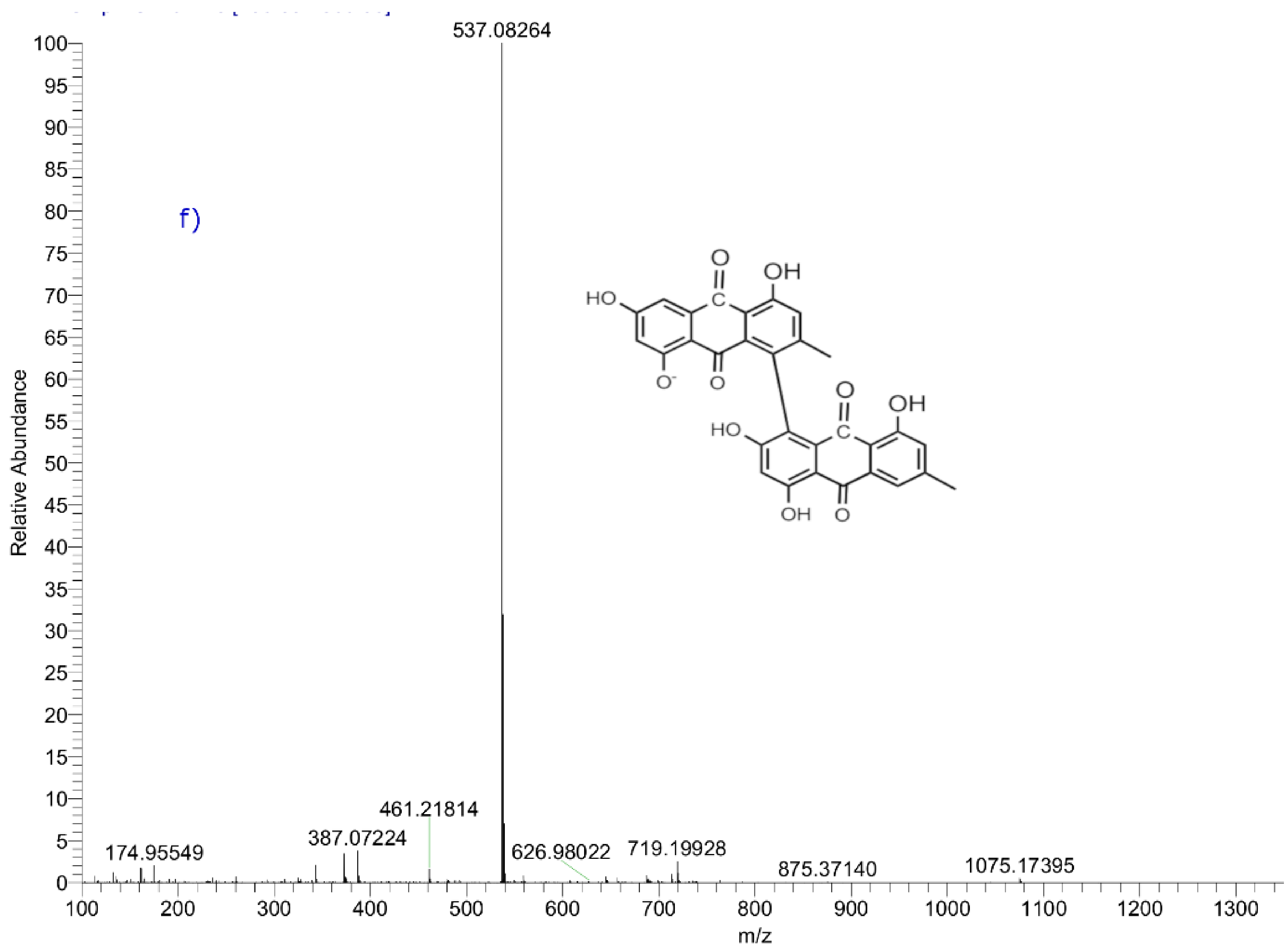

537.08264
f)
Relative Abundance
m/z
174.95549
387.07224
461.21814
626.98022
719.19928
875.37140
1075.17395
HO
OH
O
O⁻
HO
OH
OH

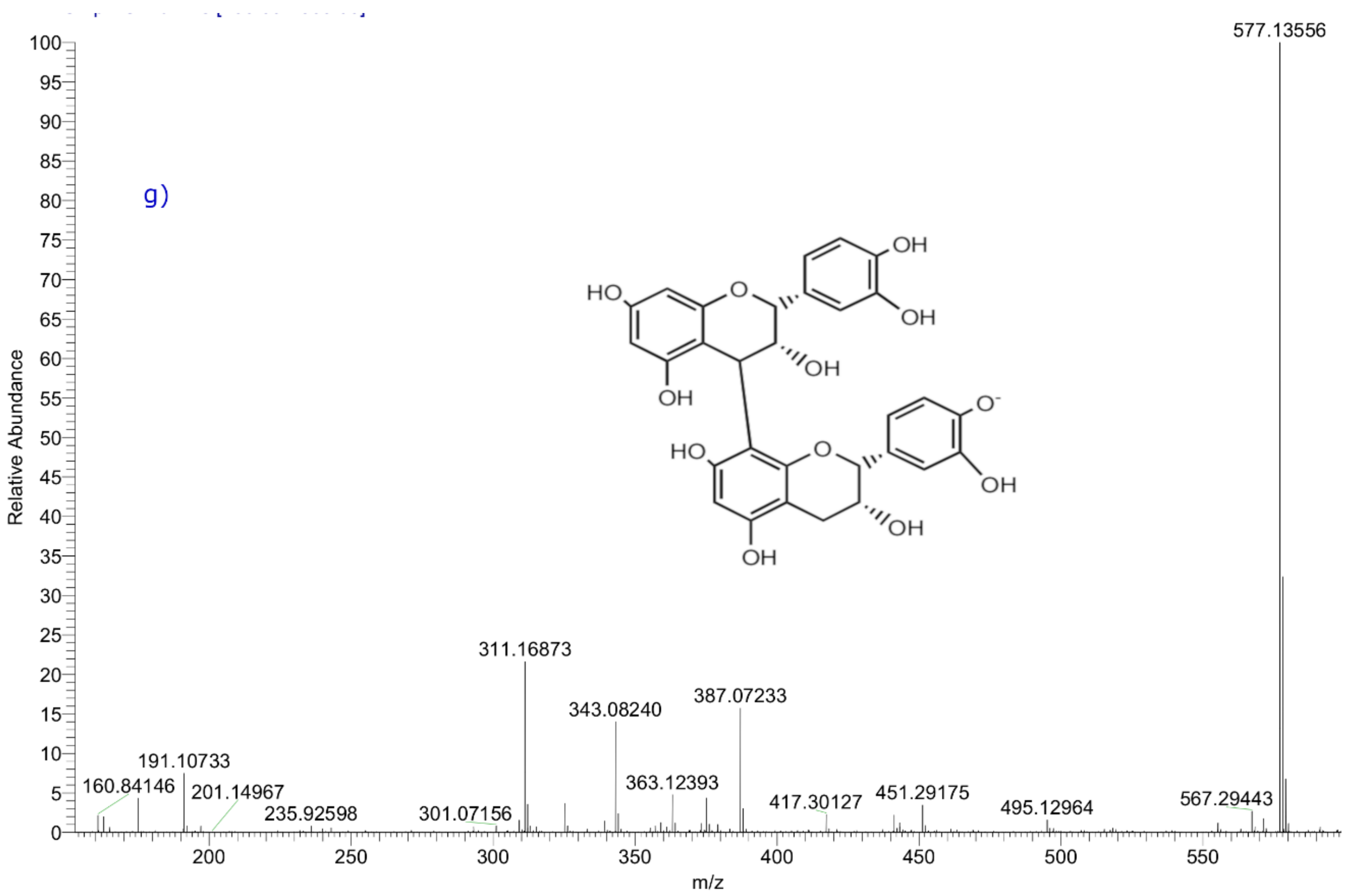

g)
577.13556
311.16873
343.08240
387.07233
191.10733
160.84146
201.14967
235.92598
301.07156
363.12393
417.30127
451.29175
495.12964
567.29443
Relative Abundance
m/z
HO
O
OH
OH
OH
OH
HO
O
O⁻
OH
OH
OH

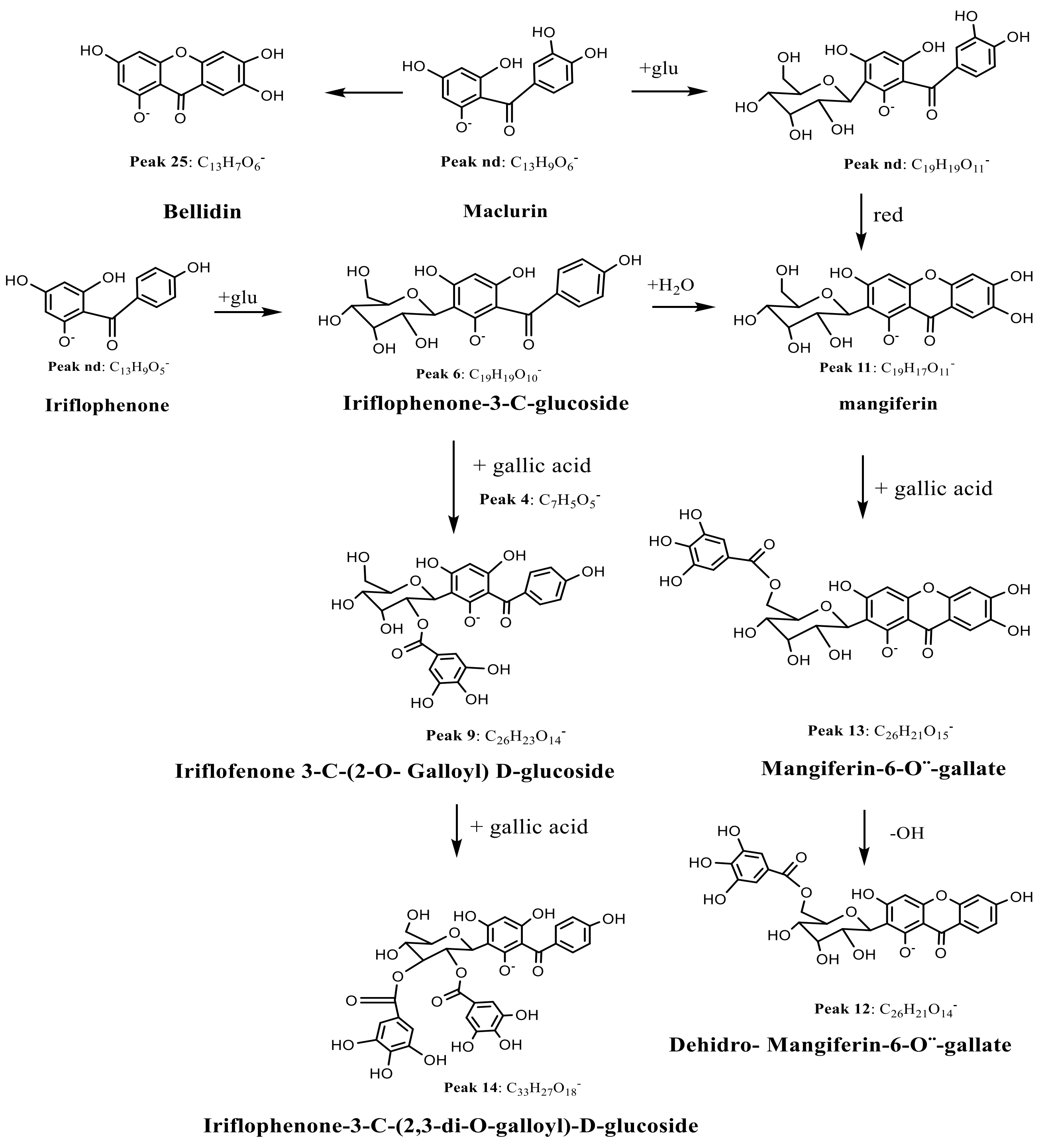

Figure S2. Biosynthetic relationship among the benzophenones detected in mango leaf extract (MLE).

The shikimate biosynthetic pathway led to maclurin, (**Figure S2**) which suffer cyclation to bellidin peak 25. Bellidin, can react by carbon–carbon coupling reactions of the activated ortho position of its 1,3 biphenol moiety with a glucose, to form mangiferin, peak 11. One galllic acid can be added to glucosa moiety of the later, to form peak 13, which loses an OH moiety to give peak 12. Another phenolic derivative, iriflophenone, can be also C-glycosilated to form peak 6, which in turn reacts with a gallic acid molecule to form peak 9, and this, in turn, forms peak 14 by esterification of an alcohol of the glucose moiety with a gallic acid molecule (**Figure S2**).

Material and Methods:

1. *Determination of antioxidant activity*

1.2. Determination of the total phenolic content (TPC)

The TPC of mango leaf extract (MLE) was determined according to the procedure reported previously [8] with some modifications. The method uses gallic acid as a reference standard. Briefly, 30 µL of sample was mixed with 150 µL of 10% Folin-Ciocalteu reagent (v/v). After 4 minutes, 120 µL of 7% sodium carbonate solution (w/v) was added, followed by 2 hours of reaction at room temperature. The absorbance of the mixture was measured at 760 nm in a microplate reader spectrophotometer (accuSkan GO UV/Vis; Fisher Scientific; PA USA). The TPC value was expressed in micrograms of Gallic Acid equivalents (µg GAE). All measurements were performed in triplicate.

1.3. DPPH Scavenging activity

The free radical scavenging capacity of the extract was determined by DPPH assay as previously described [9] with some modifications. From the different dilutions of the lyophilized extract, 12.5 µL were taken and 300 µL of the 0.1 mM DPPH reagent was added; In addition, a control (39.4 µg/mL of DPPH) and a blank were prepared. It was protected from light with aluminum foil and allowed to stand for 15 minutes. The stock solutions of DPPH were serially diluted in 96-well microplates to final concentrations of 0.8, 0.6, 0.4, 0.2 and 0.1 mM to perform a standard curve. The absorbance readings were then performed at 517 nm in a microplate reader spectrophotometer (accuSkan GO UV/Vis; Fisher Scientific; PA USA). The DPPH value was expressed in micrograms of Trolox equivalents (µg TE). All measurements were performed in triplicate.

1.4. Test of species reactive to 2-thiobarbituric acid (TBARS)

Egg yolk homogenates were used as a lipid-rich medium, according to the method previously reported [10]. Initially, 500 µL of this 10% (w/v) tissue homogenate and 100 µL of sample solutions to be tested were added. It was made up to 1 mL with distilled water, and 50 µL of AAPH solution (0.07 M) was added to induce lipid peroxidation. Then 1.5 mL of 20% TCA (pH 3.5) and 1.5 mL of 0.8% TBA (w/v) in 1.1% DDS solution (w/v) were added and the resulting mixture was vortexed and then heated at 95 °C for 60 minutes. It was then cooled to 2 °C, then 5 mL of n-butanol was added to each tube, vortexed and centrifuged at 3000 rpm for 10 minutes. The absorbance of the upper organic layer was measured at 532 nm. The calibration curve was performed with 1,1,3,3-tetraethoxypropane or malondialdehyde (MDA). In the case of the control, the same procedure was followed with the replacement of distilled water (100 µL) instead of the sample. The TBARS value was expressed in nanomoles of malondialdehyde (nM MDA). All measurements were performed in triplicate.

1.5. Ferric reduction antioxidant power test (FRAP)

It was performed according to the procedure reported previously [11] with slight modifications. Stock solutions of 300 mM acetate buffer (pH 3.6), TPTZ solution (2,4,6-tris (2-pyridyl)-s-triazine) 10 mM in 40 mM HCl and an aqueous solution of $FeCl_3x6H_2O$ were prepared 20 mM The FRAP reagent was prepared by mixing 25 mL of acetate buffer, 2.5 mL of TPTZ solution and 2.5 mL of $FeCl_3x6H_2O$ solution (10:1:1, v/v/v), then incubated at 37 °C before each test. Briefly, 25 µL of sample was incubated with 175 µL of FRAP reagent in a 96-well plate for 30 minutes in the dark and the absorbance was measured at 593 nm in a microplate reader spectrophotometer (accuSkan GO UV/Vis; Fisher Scientific; PA USA). The FRAP Value was expressed as micrograms of Trolox equivalents (µg TE), all measurements were performed in triplicate.

1.6. Radical uptake activity test (ABTS)

It was performed based on the method previously described [12] with some modifications. Initially the ABTS reagent was diluted in water to a concentration of 7 mM. The ABTS radical cation ($ABTS^{\bullet+}$) was produced by reacting the ABTS stock solution with 2.45 mM potassium persulfate ($K_2S_2O_8$) in a volume ratio of 1:1, and then incubated in the dark at room temperature for at least 12-16 hours. The ABTS stock solution was diluted with ethanol to obtain an absorbance of 0.710 ± 0.050 at 734 nm. 10 µL of diluted sample was mixed with 300 µL of $ABTS^{\bullet}$ reagent at room temperature, and the absorbance of the mixture at 734 nm was determined after 4 min in a microplate reader spectrophotometer (accuSkan GO UV/Vis; Fisher Scientific; PA USA). The ABTS value was expressed as micrograms of Trolox equivalents (µg TE), all measurements were performed in triplicate.

2. *Intestinal reactivity experiments*

2.1. Effect on the basal tone of rat ileum.

The effect on the basal tone was evaluated by cumulative concentration-response curves for the lyophilized MLE at various concentrations (0.1, 1, 10, 100 and 1000 µg/mL) with an administration interval of 5 minutes.

2.2. Spasmolytic effect in pre-contracted rat ileum

Knowing previously that musculotropic spasm can be simulated with barium chloride solutions while neurotropic spasm with acetylcholine solutions, the effects of $BaCl_2$ (80 mM) and ACh (10^{-5} M) using a single dose regimen with a pre-incubation time of 10 minutes or until the generation of a stable contractile plateau (plateau) was achieved. Then, each concentration of MLE (0.1, 1, 10, 100 and 1000 µg/mL) was approximately 5 minutes in contact with the tissue.

2.3. Antispasmodic effect in the dose-response curves of acetylcholine

In a series of experiments with the presence and absence of MLE performed an initial contraction with ACh increasing doses (10^{-10} to 10^{-4} M) at 5 min intervals between each dose, the tissue was then washed (4-5 times) and returned to its initial tension of 1 g. Subsequently, pre-incubation was performed with MLE (100 µg/mL) for 20 minutes, and subsequent contraction with ACh in increasing doses (10^{-10} to 10^{-4} M).

2.4. Role of extracellular Ca2+ influx in the relaxing response to MLE

In addition to the Tyrode solution, a calcium-free solution was prepared for this experiment. This solution had the following composition (in mM): KCl 50; NaCl 91.04; $MgCl_2$ 1.05; $NaHCO_3$ 11.87; NaH_2PO_4 0.41; glucose 5.55 and EDTA 0.1. Initially the tissue was stabilized in a normal Tyrode solution, which was then replaced with a Ca^{2+} free Tyrode solution. It was maintained only for 10 minutes of stabilization in this new solution, and ACh 10^{-5} M was added, waiting 5 minutes for the tissue to contract. Increasing amounts of $CaCl_2$ (0.1 mM; 0.3 mM; 0.6 mM, 1 mM) were added, with 5 min. intervals between each concentration. It was then washed with normal Tyrode solution to stabilize the tissue again, waiting for at least 10 minutes.

The tension was adjusted again to 1 g if necessary. Subsequently, 100 µg/mL MLE was added for 20 minutes in the same normal Tyrode solution. Then, it was replaced again with Ca^{2+} free Tyrode solution for 10 minutes and 100 µg/mL MLE was added. It contracted with ACh 10^{-5} M previously and waited 5 minutes to see the response. Finally, $CaCl_2$ (0.1 mM; 0.3 mM; 0.6 mM, 1 mM) was added.

2.5. Study of the effect of MLE and its main metabolites on oxidative damage induced by H_2O_2

After 1 hour of stabilization, the protocol was initiated by generating a contractile plateau with ACh 10^{-5} M (as 100% of maximal response), then tissue was washed with fresh tyrode solution (4-5 times) and returned to its initial tension of 1 g. Subsequently, MLE (100 µg/mL) was incubated in the organ bath for 20 minutes, then H_2O_2 (10^{-10} to 10^{-4} M) were added in cumulative concentrations. To study the role of the main metabolites of MLE on oxidative damage induced by H_2O_2 in rat ileum, mangiferin, quercetin and gallic acid were used. The ileal strips were pre-incubated with mangiferin 10^{-5} M, quercetin 10^{-5} M, or gallic acid 10^{-5} M for 20 min before the experiment. Then, tissues were exposed to cumulative concentrations of H_2O_2 (10^{-10} to 10^{-4} M). In parallel, the effect of H_2O_2 was studied in an intact portion of the ileum in the absence of MLE or its main metabolites, which served as a control.

References

1. Gómez-Caravaca, A.M.; López-Cobo, A.; Verardo, V.; Segura-Carretero, A.; Fernández-Gutiérrez, A. HPLC-DAD-q-TOF-MS as a powerful platform for the determination of phenolic and other polar compounds in the edible part

of mango and its by-products (peel, seed, and seed husk). *Electrophoresis* **2016**, *37*, 1072–1084, doi:10.1002/elps.201500439
2. Medlicott, A.P.; Thompson, A.K. Analysis of sugars and organic acids in ripening mango fruits (M*angifera indica* L. var Keitt) by high performance liquid chromatography. *Journal of the Science of Food and Agriculture* **1985**, *36*, 561–566, doi:10.1002/jsfa.2740360707. .
3. Barreto, J.C.; Trevisan, M.T.; Hull, W.E.; Erben; G.; de Brito, E.S.; Pfundstein, B.; Würtele, G.; Spiegelhalder, B.; Owen, R.W. Characterization and quantitation of polyphenolic compounds in bark, kernel, leaves, and peel of mango (*Mangifera indica* L.). *Journal of Agricultural and Food Chemistry* **2008**, *56*, 5599-5610, doi:10.1021/jf800738r.
4. Hu, K.; Dars, A.G.; Liu, Q.D.; Xie, B.J.; Sun, Z.D. Phytochemical profiling of the ripening of Chinese mango (*Mangifera indica* L.) cultivars by real-time monitoring using UPLC-ESI-QTOF-MS and its potential benefits as prebiotic ingredients. *Food Chemistry* **2018**, *256*, 171-180, doi:10.1016/j.foodchem.2018.02.014.
5. Benites, J.; Asuncion-Alvarez, H.D.; Ybanez-Julca, R.O.; Ganoza-Yupanqui, M.L.; Jacinto-Fernandez, J.J.; Reyes-De la Vega, J.B.; Zavaleta-Cruz, H.J.; Pinedo-Alcantara, A.N.; Layado-Fonseca, C.M.; Medina-Mejia, C.A., et al. Chemical composition by HPLC-ESI-QTOF-MS/MS: Estrogenic and antioxidant effects of *Mangifera indica* L. cv. "Kent" leave extracts on ovariectomized rats. *Boletin Latinoamericano y del Caribe De Plantas Medicinales y Aromaticas* **2019**, *18*, 336-346.
6. Berardini, N.; Carle, R.; Schieber, A. Characterization of gallotannins and benzophenone derivatives from mango (*Mangifera indica* L. cv. 'Tommy Atkins') peels, pulp and kernels by high-performance liquid chromatography electrospray ionization mass spectrometry. *Rapid Communications in Mass Spectrometry* **2004**, *18*, 2208-2216, doi:10.1002/rcm.1611.
7. Laulloo, S.J.; Bhowon, M.G.; Soyfoo, S.; Chua, L.S. Nutritional and Biological Evaluation of Leaves of *Mangifera indica* from Mauritius. *Journal of Chemistry* **2018**, *2018*, 1-9, doi:10.1155/2018/6869294.
8. Liu, Q.; Tang, G.Y.; Zhao, C.N.; Feng, X.L.; Xu, X.Y.; Cao, S.Y.; Meng, X.; Li, S.; Gan, R.Y.; Li, H.B. Comparison of Antioxidant Activities of Different Grape Varieties. *Molecules* **2018**, *23*, 1-17, doi:10.3390/molecules23102432.
9. Benites, J.; Ybañez-Julca, R.; Ganoza-Yupanqui, M.; Mantilla-Rodriguez, E.; Zavala, E.; Velasquez, S.; Gajardo, S.; Morales, B.; de Albuquerque, R.; Rocha, L., et al. Antioxidant effect and chemical composition of *Ananas comosus* [L.] Merr. peels from Peruvian Northern. *Boletin Latinoamericano y del Caribe De Plantas Medicinales y Aromáticas* **2019**, *18*, 578 – 586, doi:10.35588/blacpma.19.18.6.40.
10. Kulisic, T.; Radonic, A.; Katalinic, V.; Milos, M. Use of different methods for testing antioxidative activity of oregano essential oil. *Food Chemistry* **2004**, *85*, 633-640, doi:10.1016/j.foodchem.2003.07.024.
11. Rusu, M.E.; Gheldiu, A.M.; Mocan, A.; Moldovan, C.; Popa, D.S.; Tomuta, I.; Vlase, L. Process Optimization for Improved Phenolic Compounds Recovery from Walnut (*Juglans regia* L.) Septum: Phytochemical Profile and Biological Activities. *Molecules* **2018**, *23*, 1-28, doi:10.3390/molecules23112814.
12. Re, R.; Pellegrini, N.; Proteggente, A.; Pannala, A.; Yang, M.; Rice-Evans, C. Antioxidant activity applying an improved ABTS radical cation decolorization assay. *Free Radical Biology and Medicine* **1999**, *26*, 1231–1237, doi:10.1016/S0891-5849(98)00315-3.
